# Transcriptome and Zymogram Analyses Reveal a Cellobiose-Dose Related Reciprocal Regulatory Effect on Cellulase Synthesis in *Cellulosilyticum ruminicola* H1

**DOI:** 10.3389/fmicb.2017.02497

**Published:** 2017-12-12

**Authors:** Shanzhen Li, Nana Shao, Yuanming Luo, Hongcan Liu, Shichun Cai, Xiuzhu Dong

**Affiliations:** ^1^State Key Laboratory of Microbial Resources, Institute of Microbiology, Chinese Academy of Sciences, Beijing, China; ^2^State Key Laboratory of Microbial Resources, University of Chinese Academy of Sciences, Beijing, China

**Keywords:** rumen bacterium, comparative transcriptome, zymogram, cellulase synthesis, cellobiose, dose-related regulation, cellulosome, pyruvate-formate lyase

## Abstract

The rumen bacterium *Cellulosilyticum ruminicola* H1 efficiently hydrolyzes cellulose. To gain insights into the regulatory mechanisms of cellulase synthesis, comparative transcriptome analysis was conducted for cultures grown on 2% filter paper, 0.5 and 0.05% cellobiose, and 0.5% birchwood xylan. It was found that cellulose induced a majority of (hemi)cellulases, including 33 cellulases and a cellulosomal scaffoldin (1.3- to 22.7-fold); seven endoxylanases, two mannanases, and two pectatelyases (2- to 16-fold); and pyruvate formate-lyase (PFL, 1.5- to 7-fold). Noticeably, 3- and 2.5-fold increased transcription of a cellobiohydrolase and the cellulosomal scaffoldin precursor were detected in 0.05% than in 0.5% cellobiose. Consistently, 9- and 4-fold higher specific cellobiohydrolase activities were detected in the filter paper and 0.05% cellobiose culture. SDS- and native-PAGE zymograms of cellulose-enriched proteins from the filter paper culture displayed cellulase activities, and cellulolytic “complexes” were enriched from the filter paper- and 0.05% cellobiose-cultures, but not from the 0.5% cellobiose culture. LC-MS/MS identified the cellulosomal scaffoldin precursor in the “complexes” in addition to cellulase, hemicellulase, and PFL proteins. The addition of 0.5% cellobiose, but not 0.05% cellobiose remarkably inhibited strain H1 to degrade filter paper. Therefore, this work reveals a cellobiose-dose related regulatory mechanism of cellulase synthesis by lower for induction and higher for repression, which has extended our understanding of the regulation of microbial cellulase synthesis.

## Introduction

Numerous studies on microbial cellulase components and hydrolysis mechanisms have revealed 14 glycoside hydrolase (GH) clans of related 135 GH families on the basis of protein sequence similarity and folding homologs. Cellulases are generally classified as cellobiohydrolases (CBHs), which attack both reduced and non-reduced ends of cellulose and release cellobiose as the main product, and represent the key enzyme in the hydrolysis of structured celluloses, such as filter paper and Avicel; endoglucanases (EGs), which hydrolyze amorphous cellulose derivatives through an endo-action; and β-glucosidases, which hydrolyze cellobiose into glucose (Lynd et al., 2002). The three types of cellulases act synergistically to degrade cellulose into soluble oligosaccharides and glucose. Through differential transcriptome analysis, Xu et al. (2013) revealed a core set of 48 carbohydrate-active enzymes (CAZymes) required for the degradation of cellulose-containing substrates, in addition to an accessory set of 76 CAZymes required for specific non-cellulose substrates in *Clostridium cellulolyticum*.

Microbes use three strategies in plant cell wall degradation (Wilson, 2008): the free-cellulase system; the cell surface-anchored cellulase complex system, the cellulosome; and a largely unknown cell contact-dependent mode through the employment of a collection of novel cell-associated proteins (Zhou et al., 2016). In general, most aerobic cellulolytic bacteria and fungi, such as the primary industrial cellulolytic enzyme producer *Trichoderma reesei*, employ the free-cellulase system; namely, the secretion of a set of individual extracellular cellulases each with or without a carbohydrate-binding module (CBM) (Kubicek et al., 1993b; Wilson, 2008; Peterson and Nevalainen, 2012), whereas most anaerobic fibrolytic bacteria, such as *Clostridium thermocellum*, degrade cellulose by cell wall-bound cellulosomes, which usually attach to the insoluble cellulose to conduct the hydrolysis. Unlike the free system cellulases, the cellulosome comprises a characteristic scaffoldin protein, which contains multiple cohesins and a variety of cellulases and hemicellulases that are frequently embedded in a C-terminal dockerin domain to anchor to the cognate cohesins. Some cellulosome producers, such as *C. thermocellum*, also produce free cellulases (Schwarz, 2001; Schwarz et al., 2004).

In comparison with our understanding on the biochemistry of microbial cellulases, fewer studies have been conducted on the regulation of bacterial cellulolytic enzyme synthesis. It is generally accepted that bacterial cellulases, such as those in *C. thermocellum*, are synthesized constitutively (Riederer et al., 2011), but the fungal cellulases are induced by the substrate cellulose (Hammerstrom et al., 1955; Reese, 1956). However, the question arises of how the insoluble cellulose can regulate the gene expression of cellulases that occur inside cells. One hypothesis is that fungi constitutively synthesize a basal level of cellulases to attack cellulose and release smaller soluble products, which then act as inducers for a larger amount of cellulase biosynthesis (Merivuori et al., 1985). Carle-Urioste et al. (1997) reported that the promoters of *cbh1* and *egl1*, which, respectively, encode cellobiohydrolase and endoglucanase in *T. reesei*, are constitutively transcribed. Researchers have also revealed that disaccharides, such as cellobiose, lactose, and sophorose, and monosaccharides, such as L-sorbose, can serve as inducers for the *T. reesei* cellulase genes (Kubicek et al., 1993a; Morikawa et al., 1995; Schmoll and Kubicek, 2003). Because cellobiose is the major soluble end-product from the initial hydrolysis of cellulose, it is predicted to be the logical inducer for further cellulase biosynthesis. It was found that cellobiose moderately induces the cellulase gene and the enzymatic activity in fungi, *T. reesei* (Mandels and Reese, 1960; Vaheri et al., 1979), and *Aspergillus* species (Chikamatsu et al., 1999). Cellobiose clearly induced the expression of the *T. reesei cbh1* after a rather long lag period (Ilmén et al., 1997). In *Neurospora crassa*, the deletion of β-glucosidase gene allows cellobiose to induce the cellulase gene to the same level as induced by cellulose (Znameroski et al., 2012). Furthermore, lower concentrations of cellobiose (0.2–0.5 mM) induce fungal cellulases in *Thermomonospora fusca* through inhibition of the repressor CelR, which binds to a 14-base pair inverted repeat; this repeat is found in the promoter of all six cellulase genes (Spiridonov and Wilson, 1999). Collectively, the observations described above indicated that the direct product of cellulose, cellobiose, could be a potential inducer for cellulase synthesis, although this contradicts the dogma of product feedback inhibition.

*Cellulosilyticum ruminicola* H1 was isolated from yak rumen as one of the few cultured rumen cellulolytic bacteria (Cai and Dong, 2010). Both genome sequencing and zymograms indicate that strain H1 appears to use the free cellulase system by encoding an array of (hemi)cellulases carrying CBM (Cai et al., 2010), but only a few dockerin embedded cellulases (Cai et al., 2011). However, the genome carries a gene encoding a cellulosomal scaffoldin precursor, which implies that the cellulosome could be produced under unknown circumstances. Although higher specific cellulase activities were detected in corncob culture, *C. ruminicola* H1 fails to grow successively on filter paper or Avicel, unless by an interval growth on cellobiose between subcultures on cellulose. This is contrary either to the constitutive synthesis of bacterial cellulases or the substrate-induction of the fungal cellulases, and implies specific regulatory modes for the cellulase synthesis in *C. ruminicola* H1. In an attempt to reveal the regulatory mechanisms, we conducted a combination of differential transcriptome and assays of zymogram and cellulase activities for *C. ruminicola* H1 grown on filter paper, xylan, and different concentrations of cellobiose, and demonstrated that cellobiose exerts a dose-related reciprocal regulatory effect on the synthesis of cellulases or a presumable cellulosome by a lower dose for induction and a higher dose for repression. In addition, the remarkable induction of a pyruvate formate-lyase (PFL) was observed on filter paper, which implied that cellulose might even alter the metabolic shunt, most likely for efficient energy gain. The findings in this work will extend our understanding of microbial cellulase synthesis mechanism and shed light on possible improvements in cellulase production.

## Materials and methods

### Growth conditions

*C. ruminicola* H1 (CGMCC 1.5065^T^) was cultured anaerobically at 37°C under CO_2_ gas phase in the reinforced clostridium (RC) medium containing each of 0.5% (wt/vol) and 0.05% (wt/vol) cellobiose, 2% (wt/vol) filter paper (Whatman I), and 0.5% birchwood xylan. Growth experiments were conducted in three independent biological replicates. Growth in cellobiose and xylan was monitored by optical density of the culture at 600 nm (OD_600_) at 1-h (h) interval, while growth on filter paper was measured based on the increase of cellular protein in every 24 h. Cellular protein was measured by using a modified Bradford method. Briefly, culture was pelleted by centrifuge and the pellets were twice washed with 0.9% NaCl and re-suspended in 5 ml of 0.2 M NaOH. Samples were incubated in a 100°C water bath for 10 min, and after centrifuge, protein concentration in the supernatant was estimated using the Bradford assay (Bradford, 1976).

Cells used for further transcriptomic and zymogramic assays were harvested from the following cultures, 0.5% cellobiose culture at OD_600_ ≈ 0.2 (early) and OD_600_ ≈ 0.815 (late), or filter paper culture at day 2 (early, total cellular protein ≈ 41.04 μg. ml^−1^) and day 6 (late, total cellular protein ≈ 69.14 μg. ml^−1^), or 0.05% cellobiose culture at OD_600_ ≈ 0.13 (late), 0.5% xylan culture at OD_600_ ≈ 1.12 (late) (Supplementary Figure S1). Cultures are centrifuged and pelleted cells were immediately frozen in liquid nitrogen and stored at −80°C.

### Total RNA extraction for RNA-seq

Total RNA from the cellobiose and xylan cultures was extracted using Silica beads (0.5 mm diameter; Biospec) and a Mini-beadbeater (Biospec Products, Bartlesville, OK, USA) with 1 ml TRIzol reagent (Invitrogen, Carlsbad, CA, USA) according to the manufactuerer's protocol. While RNA extaction from the filter paper culture was proceeded by firstly lyzing the cells through mortar grinding in liquid nitrogen and 5 mL TRIzol® Reagent till the sample became a dried powder. The following experimental steps were the same as for cellobiose culture. NanoDrop and agarose gel electrophoresis were used to check RNA interity and concerntration (Tian et al., 2009).

### RNA sequencing and data analysis

The purified RNA samples determined by Qubit 2.0 and Agilent 100 Bioanalyzer were sequenced by means of Illumina HiSeq 2500 platform to obtain the expression libraries. Prior to data analysis, the quality of raw sequencing reads was checked by the FastQC tool (v0.10.0). The clean reads were mapped against predicted transcripts from the *C. ruminicola* H1 genome by less than two-base mismatching, using Tophat (version 2.0.8b) (Trapnell et al., 2012). The normalized expression values for each gene were calculated by the number of uniquely mapped RPKM. Differential expression gene (DEG) was determined by DESeq package (v1.5.1) (Wang et al., 2010) with raw counts of reads mapping to unique genes as input. Genes with the transcript levels having a *P* < 0.01 and fold change >2 were considered as a significant differential expression, and those with fold changes of >2 or < 0.5 were defined to be up- or down-regulated genes, respectively. The transcriptome data are deposited in NCBI database under accession number of GEO ID4957774.

Hierarchical clustering analysis of genes that showed significant differential expression in different experimental conditions was performed on the basis of RPKM values, using Cluster 3.0 with Euclidean distance as the similarity metric and complete linkage as the clustering method.

### Extracellular protein collection and (hemi)cellulase activity assays

Extracellular proteins in the spent cultures were harvested using the slightly modified method described by Cai et al. (2010). Culture supernatants of cellobiose- and filter paper-culture were collected by centrifugation at 13,400 × g at 4°C for 20 and 40 min, respectively. The supernatants were then precipitated with 80% (NH_4_)_2_SO_4_ and dissolved in 2 mL of PC buffer, and then dialyzed against the same buffer.

Protein concentrations were determined using BCA protein assay kit (Thermo Scientific, Rockford, IL). Enzymatic activities of endoglucanase, cellobiohydrolase, xylanase, mannanase, and pectinase were assayed as previously (Cai et al., 2010). One unit of enzyme activity was defined as the amount of enzyme that liberated 1 μmol of reducing sugars per minute.

### Avicel enrichment of the extracellular proteins

Avicel (Sigma, Beijing, China) was used to adhere the proteins produced by strain H1 as described by Lamed et al. (1983) with slight modifications. Supernatant was collected by centrifugation at 13,400 × g at 4°C for 40 min from a 1.5 L filter paper culture at day 6, 9, and 12, respectively. Acid-swollen Avicel was prepared by the method of Hong et al. (2008), and then added to the supernatant at final concentration of 0.1% (wt/vol). The suspension was stirred mechanically for 2 h in an ice bath, and incubated at 4°C overnight, and then centrifuged at 13,400 × g for 30 min at 4°C. Precipitation was collected and adjusted the pH to 10–11 by slowly addition of 100 mL 1% solution of trimethylamine (TEA) in ice bath. After centrifugation at 13,400 × g for 10 min at 4°C, the supernatant was collected and immediately neutralized to pH 7.0 with 10% acetic acid. Proteins in the supernatant were concentrated by slowly adding 150 mL of precooled 60% acetone, and the precipitate was dissolved in 8 mL Tris-HCl buffer (20 mM Tris-HCl, 100 mM sodium chloride, pH 7.0). By centrifugation at 13,400 × g for 30 min at 4°C, the supernatant (cellulose -bound proteins) was stored at 4°C.

### SDS-PAGE, native-PAGE, and zymogram assays of cellulose-bound proteins

Cellulose-bound proteins were run on a sodium dodecyl sulfate-polyacrylamide gel electrophoresis (SDS-PAGE) of 8% for separation and 5% for concentration. Each sample was run in two gels in parallel, by one Coomassie brilliant blue stained, and another used to generate a cellulase zymogram according to Cai et al. (2010). Modifications in manipulation were as follows, that by incubation of the gel in a renaturation buffer [25 mM Tris-HCl, 0.1% Triton X-100 (pH 7.0)] at room temperature overnight to remove the SDS in the gel, and then overlaying a 0.5% agarose gel containing 0.1% carboxymethyl cellulose (CMC-Na) on the renatured PAGE gel and incubated for 2 h at 37°C. Upon 0.1% Congo red staining for 3 min and 1 M NaCl washing, CMCase activity was estimated based on the decolored zones of the agarose gel.

Native-PAGE was used to determine the presence of cellulosome-like complex. The remaining experimental steps were the same as the SDS-PAGE.

### LC-MS/MS identification of proteins

Protein bands in Coomassie brilliant blue stained SDS-PAGE and Native-PAGE gels were subjected to in-gel trypsin digestion as described previously (Shevchenko et al., 1996). The digested peptide mixtures were identified by Thermo Scientific™ EASY-nLC™ 1000 HPLC system and Orbitrap Fusion mass spectrometer (Thermo Scientific Inc., San Jose, CA). The peptide mixtures were desalted and loaded onto homemade C18 column (75 μm ID × 15 cm, 2 μm, 100 Å) for separation. Mobile phases were composed of [A] water (0.1% formic acid) and [B] acetonitrile (0.1% formic acid). The analytical gradient was from 6 to 25% B in 55 min followed by 25 to 35% B in 15 min, then 80% B for extra 10 min. Flow rate for analytical gradients was 300 nL/min. Data was acquired on the Orbitrap Fusion MS using a resolution of 120,000 (@ 200 m/z) for full MS scans followed by HCD fragmentation and detection of the fragment ions in the Orbitrap. All MS/MS samples were analyzed using Mascot (Matrix Science, London, UK; version 2.5.1). Mascot was set up to search the *C. ruminicola* JCM 14822 genome database (Taxonomy ID: 1294025) download from the NCBI assuming the digestion enzyme trypsin. Mascot was searched with a fragment ion mass tolerance of 0.020 Da and a parent ion tolerance of 10.0 PPM.

### Determination of formic, acetic acids, and cellobiose in cultures

Formic and acetic acids in cellobiose and filter paper culture were determined by high performance liquid chromatography (HPLC). Culture supernatants were sampled, respectively at 1 h and 1 day interval during the growth in cellobiose and filter paper by centrifugation at 13,400 × g at 4°C for 20 and 40 min, respectively. One mol/L of formic and acetic acid was used as the standards. Samples were determined on a chromatographic work station (EC 2006, Elite, Dalian, China). A TSK-GEL ODS-100 V column (4.0 × 250 mm, 5 μm) was used to separate the acids at room temperature, and 0.01 M phosphoric acid solution (pH 2.5) was the mobile phase at a flow rate of 1.0 mL/min. The acids were monitored with an UV detection at 210 nm.

Cellobiose concentration in filter paper culture at day 2 and 6 was measured by high-performance anion-exchange (HPAE) chromatography using CarboPac10 analytical column at 30°C, and 0.2 M NAOH was the mobile phase at a flow rate of 1.0 mL/min.

## Results

### Comparative transcriptome analysis reveals substrate induction of the cellulase genes in rumen bacterium H1

To obtain an overview of the gene categories induced by cellulose and its hydrolytic product, cellobiose in the rumen cellulolytic bacterium H1, comparative transcriptome analysis was conducted for the cultures grown on 2% filter paper, 0.05 and 0.5% cellobiose, and 0.5% xylan. To distinguish the expression of substrate-related genes from growth phase-related genes, the transcriptomes of the 0.5% cellobiose and filter paper cultures in their early and late exponential growth phases (Supplementary Figure S1) were first compared. The high-throughput Illumina HiSeq2500 sequencing platform was used to obtain the transcriptomes and a cut-off of 2-fold difference in the transcript abundance was used to identify 1,875 differentially expressed genes (Supplementary Table S1).

**Figure 1:**
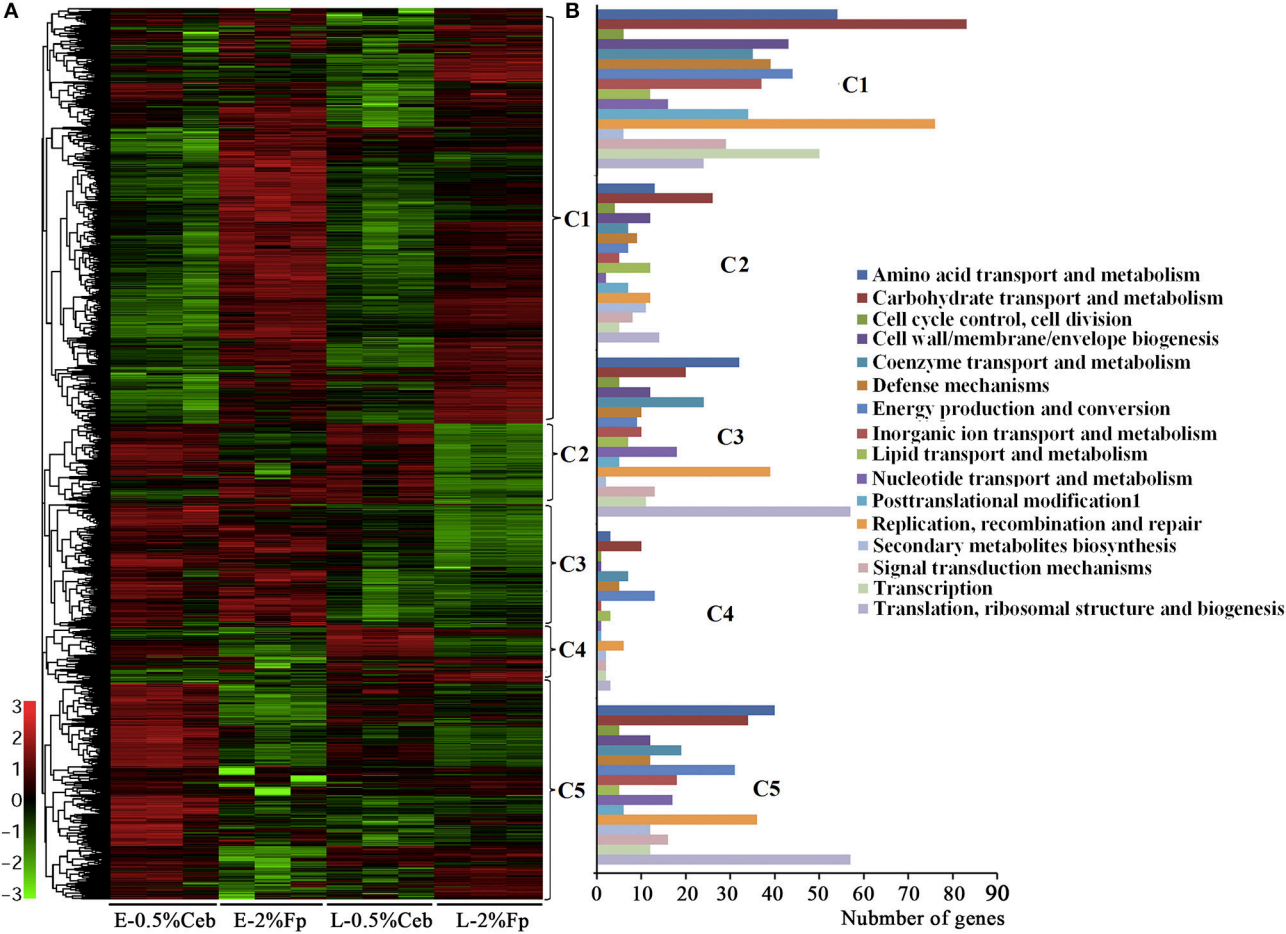
Hierarchical clustering **(A)** and functional category **(B)** analysis of differentially-transcribed genes in 0.5% cellobiose (Ceb) and 2% filter paper (Fp) in the earlier (E)- and later (L)-exponential growth phase of *Cellulosilyticum ruminicola* H1. Heat plot representation of Log2 of differential expression ratio was shown with color intensity. Green and red represent the minima and maxima abundance fold, respectively. C, Cluster.

In total, 4,202 open reading frames (ORFs) were predicted in the 4,285,341-bp genome of strain H1, with 101 genes encoding polysaccharide hydrolytic proteins. Growth phase-related expression of genes in strain H1 are defined as those that consistently show differential transcriptions in the earlier and later growth periods when grown on cellobiose and filter paper. As shown in Figure 1 and Supplementary Dataset S1, the genes with different transcription level between earlier and later growth phases in both cultures included those involved in DNA replication, transcription, and translation (Cluster 3). Remarkably increased transcription was seen in the early-exponential phase, particularly for those encoding ribosomal proteins, such as 2.9- and 3-fold higher for Crum_2681 and crum_2682, respectively. This is consistent with the rapid growth of the bacterium in the early-exponential phase. Conversely, in later growth, genes involved in coenzyme and secondary metabolisms were increased in expression, such as 220- and 100-fold increases of a pyridoxal biosynthesis lyase gene (Crum_0006) and quinolinate synthase (Crum_0999).

**Figure 2:**
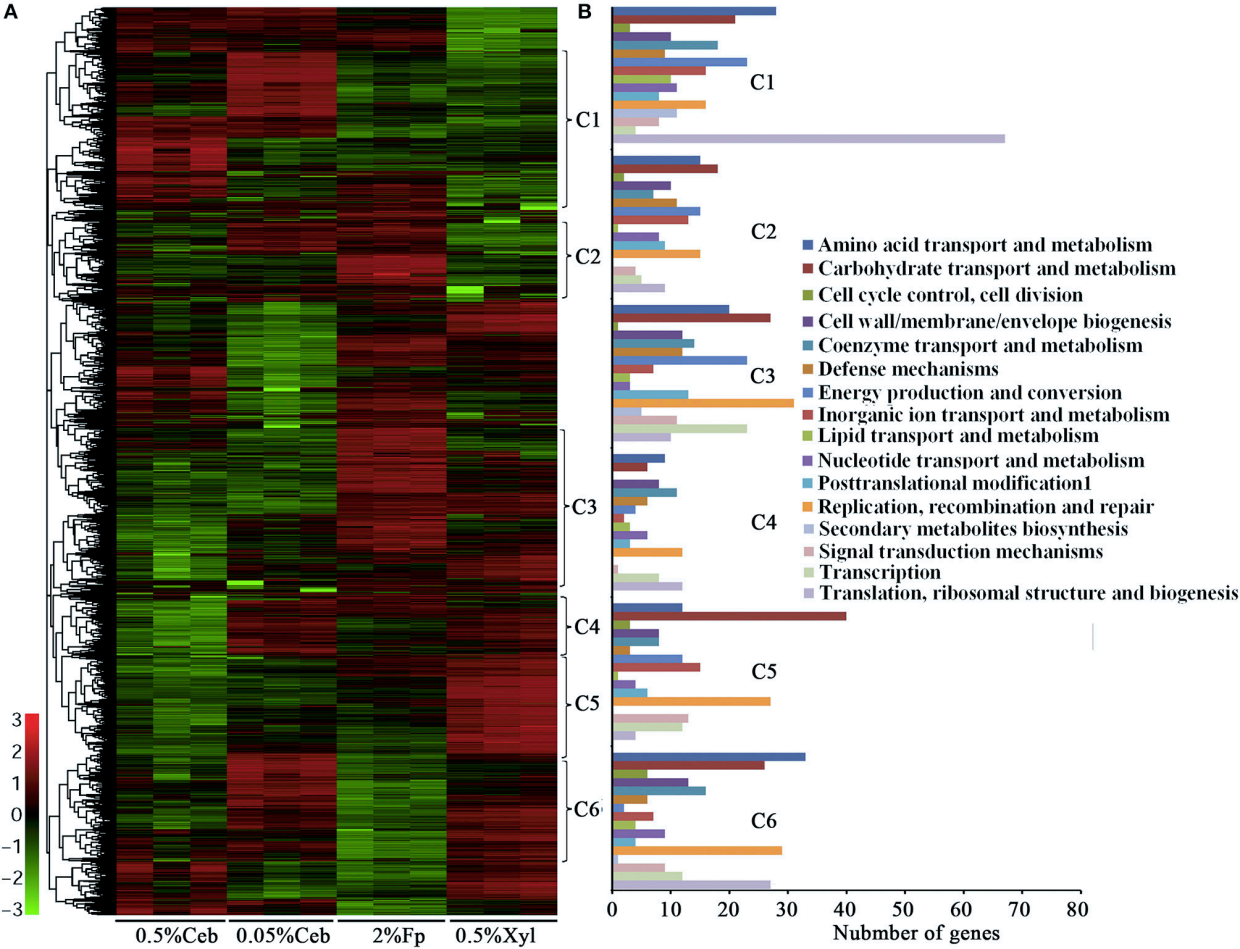
Hierarchical clustering **(A)** and functional category **(B)** analysis of differentially the transcribed genes in 0.5% and 0.05% cellobiose (Ceb), 2% filter paper (Fp), and 0.5% xylan (Xyl) of *Cellulosilyticum ruminicola* H1. Heat plot representation of Log2 of differential expression ratio was shown with color intensity. Green and red represent the minima and maxima abundance fold, respectively. C, Cluster.

Noticeably, none of the 101 genes that encode polysaccharide hydrolytic proteins, including cellulases, xylanases, mannanases, polysaccharide lyases, and esterases, were found in the growth-related transcription category. The majority of these genes were significantly increased the transcriptions in the cultures grown on filter paper and xylan compared with 0.5% cellobiose (Figure 2, Supplementary Table S2; Supplementary Dataset S2). However, a majority of the cellulases (Figure 2, Cluster 1) were revealed to have 2- to 11-fold lower transcriptions when grown on 0.5% cellobiose than on 2% filter paper. This suggests that transcription of the cellulase genes was induced by the substrate cellulose, but suppressed by the hydrolytic product cellobiose. While in the filter paper culture, most cellulase genes also were found in a growth phase-related transcription mode, such as 7.0- and 11.1-fold higher transcriptions of the cellobiohydrolase gene Crum_2625 and the endoglucanase gene Crum_1559 found in early than in late exponential growth. This implies that other factors, such as the accumulation of cellobiose or the fermentation products during growth, may affect the transcription. Of the 52 differentially transcribed (hemi)cellulase genes, 32 were increased in transcript abundance by 1.3- to 22.7-fold in filter paper- and xylan-cultures (Supplementary Table S2, Supplementary Dataset S2). These were generally efficiently transcribed with >100–1,400 RPKM values; and Crum_2624 and Crum_2625, which, respectively, encode endo- and exo-glucanases, were among the most transcribed categories and indicates their primary cellulolytic roles in strain H1. It is worthy of noting that Crum_1558, a gene encoding a cellulosomal scaffolding protein precursor, also showed >5-fold elevated transcription on filter paper. Although some cellulase genes appeared to be induced by 0.5% cellobiose, most of them were poorly transcribed. Therefore, cellulases in rumen bacterium H1 are defined as substrate-induced enzymes. Interestingly, some hemicellulases, such as a xylanase gene cluster (Crum_2062–2063) and a mannanase gene (Crum_2311) showed increased transcriptions in the filter paper compared with cellobiose, even on xylan (Figure 2). Remarkably, the transcript abundance of Crum_2311 was the highest in the transcriptome (Figure 2, Supplementary Table S2), which suggests that cellulose also induces hemicellulases.

As shown in Supplementary Dataset S2, two genes (Crum_1416 and Crum_1417) that encode the LacI family regulator were increased >3-fold and a LytTR gene (Crum_2623), which is linked with the endoglucanase and cellobiohydrolase genes (Crum_2624 and _2625), increased the transcription by 1.5- and 11.5-fold, respectively, in 2% filter paper and 0.5% birchwood xylan compared with 0.5% cellobiose. This suggests that they are involved in the regulation of cellulase induction. Noticeably, the RNA chaperone Hfq gene (Crum_1151) was upregulated 4- and 8.5-fold in the 2% filter paper and 0.5% birchwood xylan compared with 0.5% cellobiose, which suggests that post-transcriptional regulation may exert a role in cellulose-induced cellulase synthesis. The genome of strain H1 encodes >200 ABC transporters. The transcription of an ABC transporter system (Crum_1413–1415), which is linked to a LacI regulator (Crum_1416–1417), was increased 7-fold transcription in 2% filter paper; in particular, the transcription of a cyclodextrin transporter (Crum_1415) was increased 14-fold in birchwood xylan, which suggests that they play a role in the uptake of trace amounts of cellobiose and oligosaccharides. In addition, the transcription of a cytochrome C2 gene (Crum_0809) was increased 4-fold in the filter paper than in 0.5% cellobiose, but was not different to the transcription in birchwood xylan, whereas the chaperonin GroEL gene also increased transcription (4.8- to 8.8-fold) in the (hemi)celluloses than in 0.5% cellobiose, which indicated that the bacterium was also stressed by the cellulase-induced conditions.

### Enzymatic activities and zymogram assays validate substrate-induced synthesis of cellulases and hemicellulases in strain H1

To determine that substrate induction of the transcription of cellulase genes also affects the protein level, we further compared the cellulase activities of the 0.5% cellobiose and 2% filter paper cultures. Approximately 10-fold higher activities of both cellobiohydrolase and endoglucanase were detected in the filter paper culture than the 0.5% cellobiose culture in the early growth phase (Supplementary Table S3). In addition, remarkably higher enzymatic activities of mannanase and pectinase, but not xylanase, were determined in the filter paper culture (Supplementary Table S3), thereby confirming that cellulose induces the three types of enzymes.

**Figure 3:**
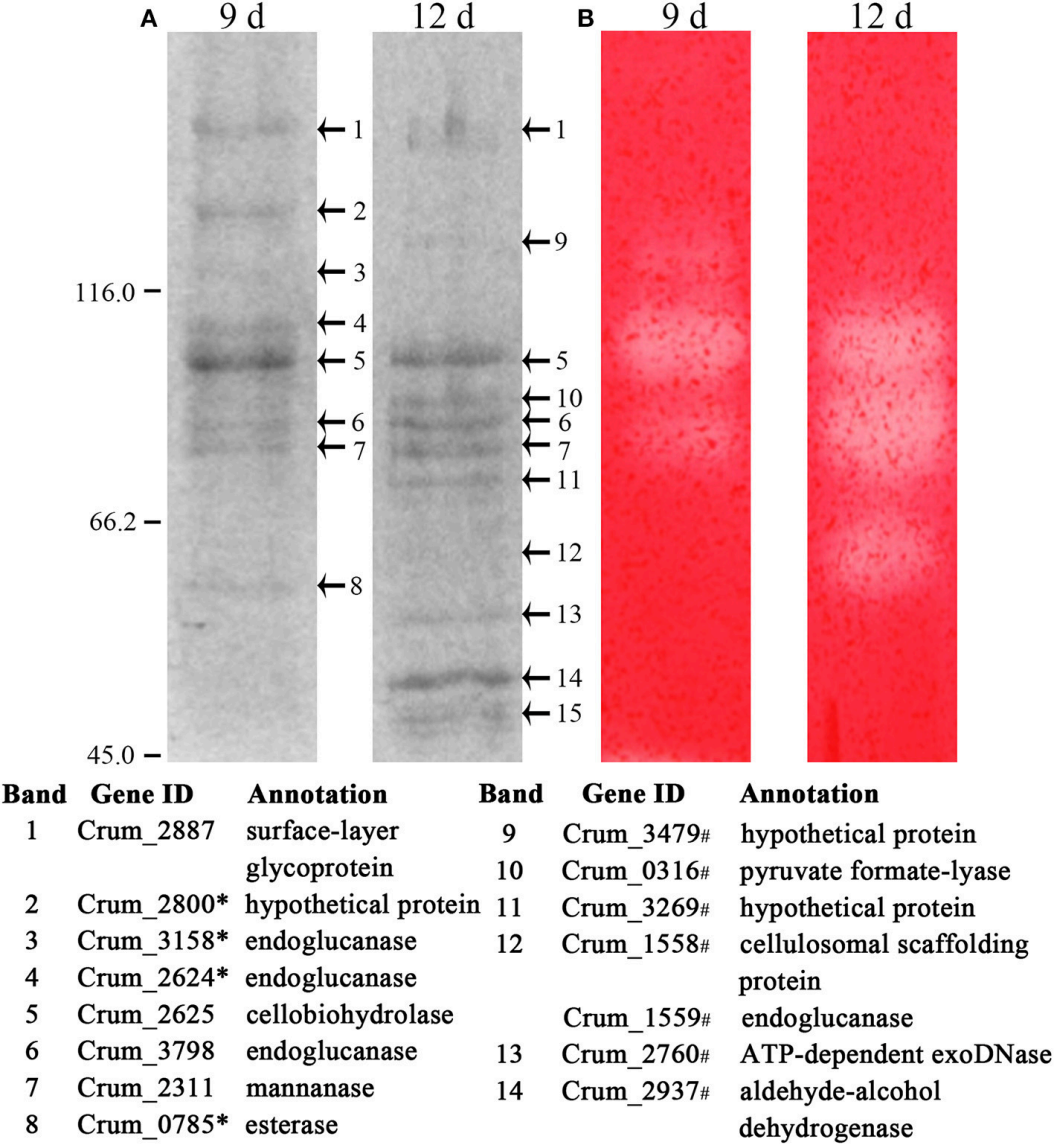
Identification of cellulose-bound proteins enriched from the spent filter paper culture. **(A)** Cellulose-bound proteins were electrophoresed on SDS-PAGE (8% for separation and 5% for concentration), and visualized by Coomassie brilliant blue staining. Arrowheads point to the protein bands that are identified by LC-MS/MS and the identification with a corresponding number to each band is listed beneath the gel. **(B)** CMCase activity of cellulose-bound proteins in SDS-PAGE is assayed by an overlaid agarose gel containing CMC and Congo red staining. The culture days when enriched proteins are indicated on the lane top and molecular masses in kilodaltons are indicated on the left. ^*^ and # refers to the proteins identified only in day 9 and day 12 cultures, respectively. A locus without a mark refers to the proteins identified in both cultures. The representative images from Supplementary Figure S2 are shown here.

To identify the exact proteins that contributed to the higher cellulase activities in filter paper culture, we harvested the extracellular proteins from the culture on days 6, 9, and 12. Given that most of the cellulases of strain H1 embed a CBM (Cai et al., 2010), acid-swollen Avicel was used to absorb the proteins. Cellulose-bound proteins were then separated on SDS-PAGE and a zymogram was generated based on *in gel* enzymatic activity assayed after the proteins were renatured. As shown in Figure 3A and Supplementary Figure S2, cellulose adhered more and different proteins from day 12- than day 9-filter paper cultures, and CMCase activity was determined from four and five protein bands from day 9- and day 12-cultures (Figure 3B), respectively. Next, the SDS-PAGE protein bands were identified by LC-MS/MS, and those included the cellulose-induced proteins as endoglucanase (Crum_1558, _1559, _2624, _3158, and _3798), cellobiohydrolase (Crum_2625), and mannanase (Crum_2311). Proteins from day 6 culture were not included for enzymatic assay or LC-MS/MS identification because of very few and faint bands in the sample. Therefore, a combination of zymogram assay and protein identification demonstrated that the substrate cellulose induced the synthesis of cellulases in *C. ruminicola* H1.

Noticeably, acid-swollen Avicel also adhered non-cellulolytic proteins, and even some cytoplasmic proteins, such as surface proteins, PFL, protease, and DNase, and none of these carry a CBM. This could be a result of to their abundance and release by cell lysis.

### Lower cellobiose appears to induce cellulase synthesis

Although the comparative transcriptome showed that the cellulase genes were induced on filter paper, it is hard to imagine that insoluble cellulose exerts a direct action on cytoplasmic events. While the direct hydrolytic product of cellulose, cellobiose at lower than 0.5% could be present in the filter paper culture. Approximately 0.0019 and 0.025% cellobiose was measured in filter paper cultures at day 2 and 6, respectively, using HPAE chromatography. Next, comparative transcriptome analysis was performed for 0.05 and 0.5% cellobiose-culture. As shown in Figure 2, Supplementary Table S2, and Dataset S2, some cellulase genes showed increased transcriptions in 0.05% than in 0.5% cellobiose, for example, 3.4-, 2.51-, and 2.87-fold elevated transcriptions were found for the endoglucanase genes Crum_1236, Crum_1558, and the cellobiohydrolase gene Crum_2625, respectively. Consistently, approximate 3-fold higher cellobiohydrolase activity was detected in the 0.05% cellobiose culture than in the 0.5% cellobiose culture (Supplementary Table S3). Therefore, cellobiose appears to exert a dose-related reverse regulatory action on the transcription of cellulase genes, whereby a lower concentration results in induction and a higher concentration results in repression, respectively. The cellobiose dose-related effect could also explain the reduced transcription of cellulase genes in the later-phase growth in filter paper (Figure 1, Cluster 1).

**Figure 4:**
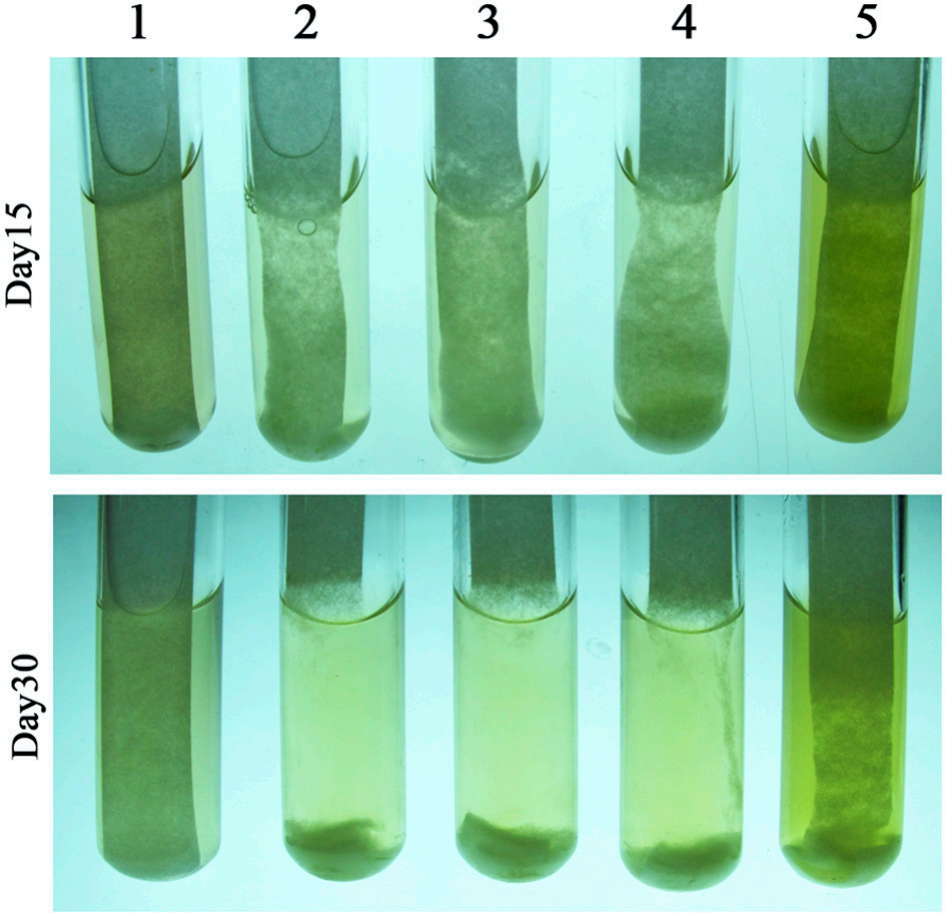
Effect of cellobiose on filter paper degradation by *Cellulosilyticum ruminicola* H1. The RC broth was used as the basic medium and with a piece of filter paper adhered to the tube wall. Cellobiose and glucose were added as the indicated final concentrations. After strain H1 inoculated the cultures were incubated at 37°C. 1, Filter paper; 2, filter paper inoculated with strain H1; 3–5, strain H1 inoculated filter paper with 0.05% glucose, 0.05% cellobiose, and 0.5% cellobiose, respectively.

To confirm the dose-related regulatory effect of cellobiose in cellulase synthesis, 0.05 and 0.5% cellobiose were, respectively, added into the filter paper cultures, in addition to 0.05% glucose. As shown in Figure 4, although 0.05% cellobiose did not appear to accelerate the degradation, 0.5% cellobiose markedly retarded the degradation of filter paper, which validates the dose-related effect of cellobiose in cellulase synthesis of the rumen bacterium H1. Unexpectedly, glucose appears to slightly inhibit filter paper hydrolysis, although the bacterium does not grow on glucose, which implied that carbon catabolite repression (CCR) might be involved in cellulose degradation.

### Either cellulose or lower-concentration cellobiose induces a tentative cellulosome-like complex

**Figure 5:**
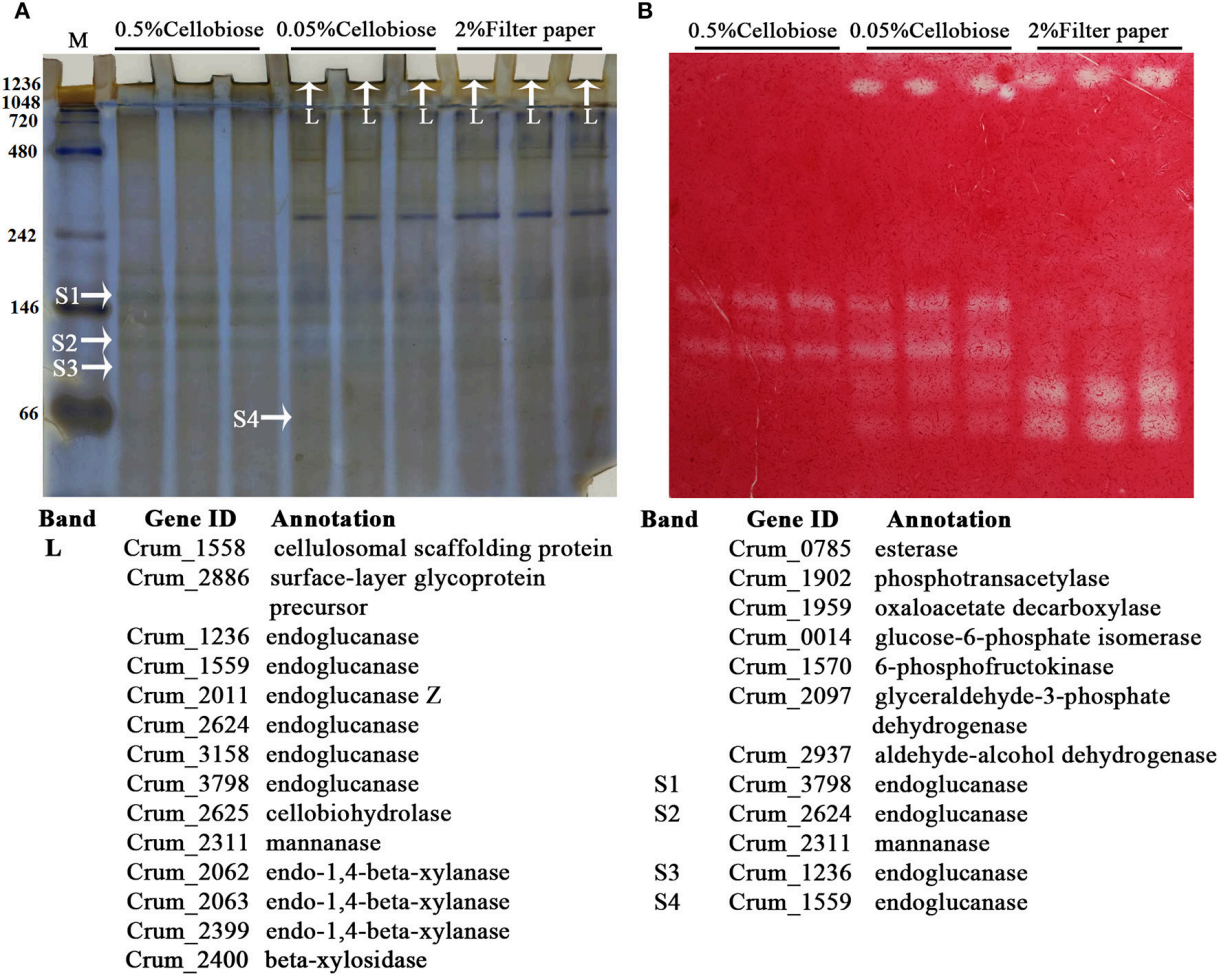
The zymogram and identification of cellulose-bound protein complexes in the spent cultures of 0.05% cellobiose and filter paper. **(A)** Cellulose-bound proteins were separated on a native PAGE (8%) and visualized by Coomassie brilliant blue staining. Arrowheads point to the LC-MS/MS identified proteins that are listed beneath the gel. L, larger complex; S, small protein. **(B)** CMCase activity of the cellulose-bound proteins in native-PAGE was assayed by an overlaid agarose gel containing CMC and Congo red staining. The molecular masses in kilodaltons are indicated on the left.

Similar to other fibrolytic anaerobes, strain H1 also contains a gene encoding a cellulosomal scaffoldin (Crum_1558), and a clustered gene encoding an endoglucanase (Crum_1559) with a dockerin domain. This suggests that strain H1 may produce a cellulosome, although previous work did not isolate this complex from the corncob culture (Cai et al., 2010). Transcriptome analysis also showed 5- and 2-fold higher transcript abundances of Crum_1558 and Crum_1559 in the filter paper and 0.05% cellobiose than in 0.5% cellobiose culture, respectively. This suggested that the cellulosome could be synthesized on cellulose and lower-concentration cellobiose. We then used native-PAGE to isolate large protein complexes from the 0.05% cellobiose- and filter paper-culture in their late-exponential phases by including 0.5% cellobiose culture in parallel. As shown in Figure 5, the presumed protein complexes, with a molecular mass >1,236 kDa, were found in both the 0.05% cellobiose- and filter paper-cultures, which were stacked in the native-PAGE loading wells and showed detectable CMCase activity. However, similar large protein complexes were not detected in the 0.5% cellobiose culture. In addition, proteins of < 200 kDa with CMCase activity were also found in all the three cultures. However, larger CMCase proteins (Figure 5, bands S1 and S2) were only found in cellobiose cultures, irrespective of concentrations, and the smaller proteins (Figure 5, bands S3 and S4) were present in both the 0.05% cellobiose and filter paper cultures. This suggests that a lower concentration of cellobiose, similar to filter paper, plays an inductive role in synthesis of cellulase or cellulosome-like complexes, which provided further evidence that a low concentration of cellobiose was the inherent inducer of cellulose for cellulase induction. Subsequently, LC-MS/MS identification of the components of the “complex” revealed the cellulosomal scaffolding protein precursor (Crum_1558) and endoglucanases, including Crum_1559, cellobiohydrolase Crum_2625, and hemicellulases such as mannanase, xylanase, and esterase (Figure 5). The smaller proteins (Figure 5A, bands S1, S2, S3, and S4) were identified as endoglucanase (Crum_3798, Crum_2624, and Crum_1599), and mannanase (Crum_2311). In addition, a glycoprotein-like surface-layer glycoprotein precursor was also included in the complex.

### Pyruvate-formate lyase synthesis is promoted by cellulose but suppressed by cellobiose

Cellobiose, but not glucose, is used as the carbon source by strain H1 (Cai and Dong, 2010), so it could be phosphorylated by cellobiose phosphorylase before being channeled to glycolysis through the Embden-Meyerhof-Parnas (EMP pathway). A cellobiose phosphorylase gene (Crum_1590) and most of the genes involved in the EMP were induced by cellobiose, particularly by 0.05% cellobiose (Supplementary Figure S3 and Table S2). Differential transcriptomics also showed that the transcript abundance of Crum_0316, a PFL, was not only the highest (RPKM = 59031), but was also 5-fold higher in filter paper culture than in the cultures of 0.5 and 0.05% cellobiose (Figure 2, Supplementary Dataset 2), which suggested that cellulose induces PFL. PFLs, also known as formate acetyl transferases, are the main enzymes involved in formate synthesis during the conversion of pyruvate to acetyl-CoA in various organisms, including the cellulolytic clostridia. A PFL-activating protein (Crum_0315), which activates PFL by acting on the amino acid residues of PFL catalytic site to form glycyl radical, was also markedly upregulated in the filter paper culture (Figure 2, Supplementary Dataset S2).

**Figure 6:**
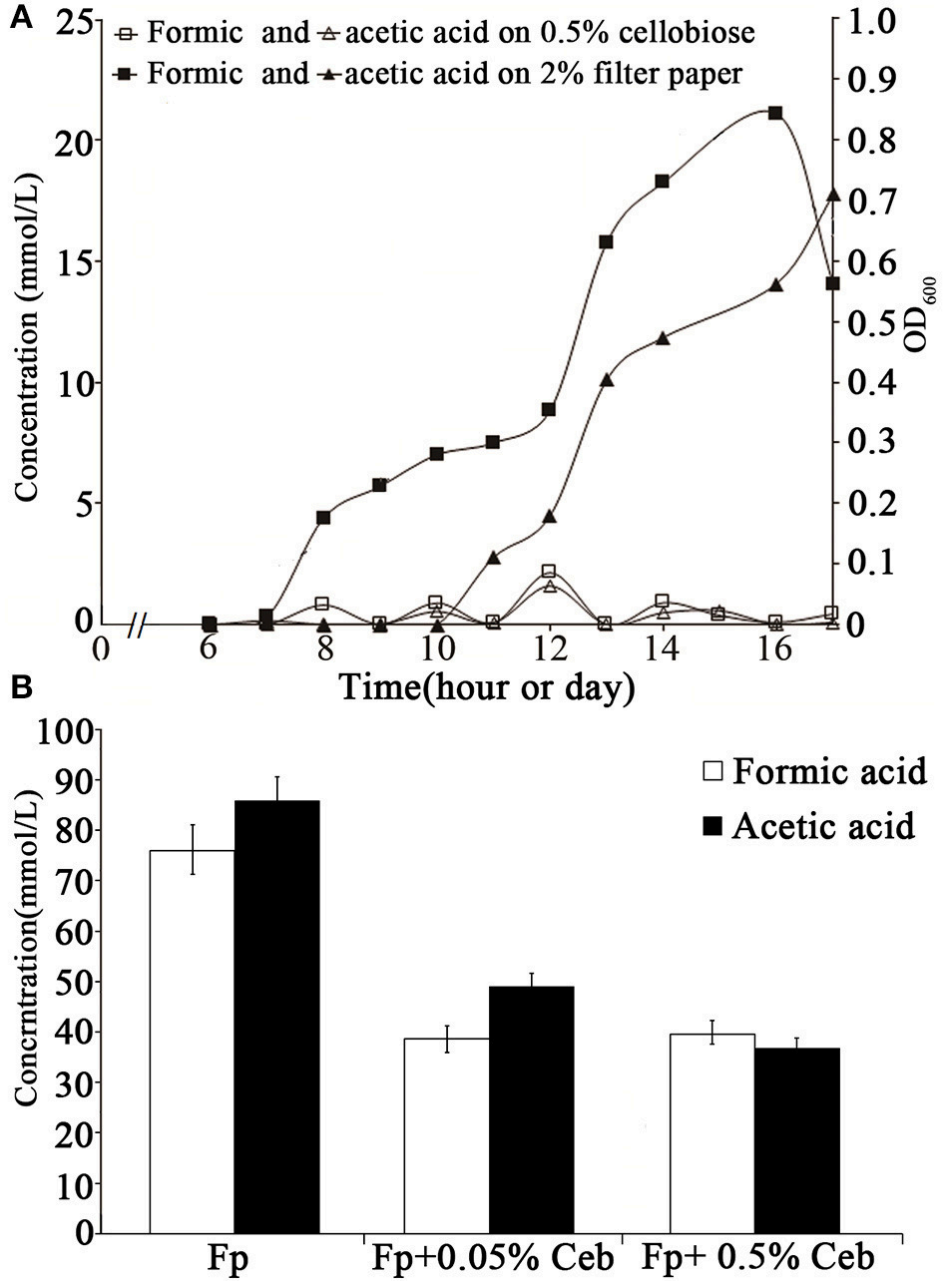
Accumulation of formic and acetic acids in the cultures in cellobiose and filter paper. **(A)** The growth and sampling times of the cellobiose- and filter paper- cultures are shown in hours and days, respectively. **(B)** Formic and acetic acid yields were measured in filter paper culture (Fp) with cellobiose (Ceb) on day 30 (Figure 4). The average was obtained from three batches of cultures and the standard deviation is shown.

To determine the cellulose-induced PFL activity, HPLC was used to determine the accumulation of formic and acetic acids in the cultures of 0.5% cellobiose and filter paper. It was found that ~20 mM formic acid and 18 mM acetic acid accumulated in the filter paper culture at the end of growth. Although a high abundance of the *pfl* transcript was also detected in the cellobiose cultures (RPKM-values: 13,986 in 0.5% and 9,132 in 0.05%), the two acids (< 2 mM) were hardly detected in either 0.05 or 0.5% cellobiose (Figure 6A). To test the inhibitory effect of cellobiose on PFL synthesis, acid accumulation was assayed in the filter paper culture with addition of 0.05% or 0.5% cellobiose (Figure 4), but approximately half yield of formic acid yield was detected than in the filter paper cultures at the end of fermentation (Figure 6B), thereby confirming the inhibitory effect of cellobiose on PFL synthesis. Although the mechanism remains elusive, it was confirmed that the bacterium employed different metabolic shunts when growing on cellulose, probably to gain more energy via PFL-mediated pyruvate cleavage to acetyl-CoA and formate.

## Discussion

Through a combination of differential transcriptome and zymogram assays, this study determined that the rumen cellulolytic bacterium *C. ruminicola* H1 employs a mechanism of substrate cellulose, specifically, trace contents of its hydrolytic product, cellobiose, induced the synthesis of cellulases, in particular cellobiohydrolase, and a presumable cellulosome, an efficient fibrolytic protein complex. Remarkably, cellobiose exerts a dose-related reverse regulatory effect on the rumen bacterial cellulase synthesis, whereby a lower concentration leads to induction and a higher concentration leads to repression. In addition, PFL synthesis, which mediates pyruvate cleavage to formic acid and acetyl-CoA, was promoted by cellulose, but suppressed by cellobiose; this process may enable the bacterium to gain more ATP from the increased acetyl-CoA.

Unlike *C. thermocellum*, in which expression of the genes encoding endoglycanases GH5 and GH9, but not the cellobiohydrolase GH48, are growth rate-dependent (Riederer et al., 2011), the transcription of polysaccharide hydrolases in the rumen bacterium H1 remain unchanged during growth in cellobiose (Supplementary Dataset S1). However, the reduced transcription of cellulase genes in the cellulose culture may result from the high accumulation of cellobiose in late growth stages. Therefore, *C. ruminicola* H1 primarily employs a mechanism of substrate-dependent cellulase synthesis. Although strain H1 carries a gene encoding the cellulosome characteristic scaffoldin, no cellulosome was identified in the cells that were grown in 0.5% cellobiose or complex hemicellulose (Cai et al., 2010, 2011). However, in this study, the proposed cellulolytic protein “complexes” have been found in the cells cultured either in 2% filter paper or 0.05% cellobiose (Figure 5). Noticeably, although both 0.05% cellobiose and filter paper both induced these fibrolytic complexes, cellobiose induced divergent categories of cellulases, irrespective of the concentration, from those induced by filter paper. For example, the two specifically induced by cellobiose, Crum_3798 and Crum_2624, all carry CBMs at the C-terminus, an indication of free enzymes. However, the filter paper-induced gene Crum_1559 carries a dockerin I module, which indicates that it is a component of cellulosome. The substrate-dependent mechanism could be also applied to cellulose or trace cellobiose induction on the proposed cellulolytic protein complex.

Based on the data, we predicted that the rumen bacterium H1 uses the basal cellulase to hydrolyze cellulose and generate trace cellobiose to initiate an induction of large synthesis of cellobiohydrolase and the predicted cellulosome. Similarly, in the fibrolytic fungus *N. crassa*, cellobiose, and its derivatives, such as cellobiose, cellotriose, and cellotetraose, efficiently induce the cellulase gene expression (Znameroski et al., 2012). Whereas, in *Aspergillus niger*, higher transcriptions of xylanolytic genes were found in xylan or xylose than in glucose, but >1 mM xylose represses the expression of xylanolytic genes, which are mediated by the carbon catabolite repressor (de Vries and Visser, 1999; de Vries et al., 1999). It appears that bacteria use approximate 10-fold lower cellobiose (0.05%) than fungi for cellulase induction. It has also been found that other polysaccharide-derived oligosaccharides, such as laminaribiose, but not the direct product cellobiose, induce cellulase gene expression in the fibrolytic bacterium *C. thermocellum* (Newcomb et al., 2007). A LacI family transcriptional regulator, GlyR3 (Cthe2808), was determined to implement the regulation of laminaribiose induction on cellulase gene expression (Rydzak et al., 2012). Strain H1 also possesses genes that encode LacI family regulators, which could play a role in regulation of the (hemi)cellulases synthesis.

Additionally, cellulose appears to induce the rumen bacterium H1 to use an alternative carbon metabolic shunt, as several PFL genes are markedly increased in expressions in the filter paper culture and pyruvate-formate lyase activity does elevate (Figure 2, Supplementary Datasets S2). However, cellobiose represses pyruvate-formate lyase activity, irrespective of the concentration, yet the mechanism remains unknown. Although the Avicel-induction of PFL-related proteins is also observed in *C. thermocellum*, formate was not detected in the cellulose culture (Riederer et al., 2011). PFLs cleave pyruvate and produce formic acid and acetyl-CoA and ATP. This could be an energy-economic approach for cellulolytic bacteria on the recalcitrant substrate cellulose.

In conclusion, the findings of this work reveal not only a cellobiose-dose-related reverse regulation of cellulase synthesis in a rumen bacterium, but also the cellulose-mediated induction of an alternative carbon metabolism shunt.

## Author contributions

SL and NS performed the experiments; YL performed mass spectrometric identification of peptides; HL run HPLC; SC helped for enzymatic assay and XD designed the project and wrote the manuscript text. All authors read and approved the final manuscript.

### Conflict of interest statement

The authors declare that the research was conducted in the absence of any commercial or financial relationships that could be construed as a potential conflict of interest.
