# Supplementary material for: Transcriptome and Zymogram Analyses Reveal a Cellobiose-Dose Related Reciprocal Regulatory Effect on Cellulase Synthesis in Cellulosilyticum ruminicola H1
Source: Front Microbiol. 2017 Dec 12;8:2497. doi: 10.3389/fmicb.2017.02497 (PMC5733062; doi:10.3389/fmicb.2017.02497)
Supplement: Supplementary file 3 [file Table1.doc]

**Transcriptome and zymogram analyses reveal a cellobiose-dose related reciprocal regulatory effect on cellulase synthesis in Cellulosilyticum ruminicola H1**

Shanzhen Li^1,2,#^, Nana Shao^1,2,#^, Yuanming Luo^1^, Hongcan Liu^1^, Shichun Cai^1^, Xiuzhu Dong^1*^

^1^State Key Laboratory of Microbial Resources, Institute of Microbiology, Chinese Academy of Sciences. Beijing 100101, P. R. China

^2^University of Chinese Academy of Sciences, Beijing 100049, China

Corresponding author: Xiuzhu Dong, No.1 Beichen West Road, Chaoyang District, Beijing 100101, P. R. China

Tel: +86 10 64807413

E-mail: [dongxz@im.ac.cn](mailto:dongxz@im.ac.cn)

^#^These authors contributed equally to this work

**Supplementary Table S1-S3**

**Supplementary Figure S1-S3**

**Supplementary tables**

**Supplementary Table S1** Statistics of differentially transcribed genes on different substrates at different growth phases.

| DEG Set | Up-regulated | Down-regulated |
| --- | --- | --- |
| Late- vs. early-growth: |  |  |
| 2% filter paper | 524 | 512 |
| 0.5% cellobiose | 377 | 472 |
| Substrates at late growth： |  |  |
| 0.5% cellobiose vs. 2% filter paper | 702 | 765 |
| 0.5% cellobiose vs. 0.05% cellobiose | 531 | 770 |
| 0.5% cellobiose vs. 0.5% xylan | 736 | 801 |
| 0.05% cellobiose vs. 0.5% xylan | 743 | 829 |
| 0.05% cellobiose vs. 2% filter paper | 842 | 818 |
| 2% filter paper vs. 0.5% xylan | 702 | 792 |

Abbreviation: DEG, differentially expressed genes.

**Supplementary Table S2**. Transcript levels of the polysaccharides hydrolytic proteins of strain H1 that grew in different carbohydrates

| **Gene ID** | **Annotation** | **0.5% cellobiose** | **2% filter paper** | **0.05% cellobiose** | **0.5%**  **xylan** |
| --- | --- | --- | --- | --- | --- |
| Crum_0053 | glucosidase | 151 | 43 | 332 | 268 |
| Crum_0057 | alpha-L-fucosidase | 25 | 11 | 27 | 13 |
| Crum_0086 | endo-1,4-beta-xylanase | 31 | 66 | 7 | 24 |
| Crum_0087 | endo-1,4-beta-xylanase/GH 10 | 32 | 66 | 5 | 17 |
| Crum_0089 | endoxylanase | 64 | 844 | 38 | 1798 |
| Crum_0097 | glycosyl hydrolase family 88 | 7 | 10 | 15 | 14 |
| Crum_0100 | esterase | 25 | 32 | 40 | 92 |
| Crum_0110 | esterase | 54 | 79 | 135 | 77 |
| Crum_0132 | glycosyl hydrolase, family 16 | 26 | 20 | 25 | 77 |
| Crum_0274 | glycosyl hydrolase family 4 | 3 | 4 | 5 | 2 |
| Crum_0321 | gh18 chitinase-like domain | 83 | 125 | 91 | 252 |
| Crum_0388 | endoglucanase | 3 | 5 | 6 | 6 |
| Crum_0396 | glycoside hydrolase | 3 | 8 | 3 | 5 |
| Crum_0397 | glycoside hydrolase | 3 | 10 | 2 | 5 |
| Crum_0398 | glycoside hydrolase | 4 | 11 | 2 | 5 |
| Crum_0437 | endo-1,4-beta-xylanase A | 35 | 26 | 44 | 8 |
| Crum_0438 | galactosidase | 112 | 113 | 41 | 354 |
| Crum_0439 | galactosidase | 288 | 186 | 70 | 370 |
| Crum_0471 | pectin methylesterase | 14 | 206 | 16 | 18 |
| Crum_0475 | polygalacturonase | 11 | 37 | 25 | 27 |
| Crum_0625 | endoglucanase | 218 | 28 | 204 | 328 |
| Crum_0626 | endoglucanase | 208 | 27 | 219 | 261 |
| Crum_0754 | endoxylanase | 4 | 1 | 1 | 44 |
| Crum_0755 | endoxylanase | 3 | 1 | 1 | 31 |
| Crum_0785 | esterase | 67 | 189 | 38 | 203 |
| Crum_0825 | polysaccharide deacetylase | 1 | 4 | 1 | 0 |
| Crum_0829 | alpha-galactosidase | 53 | 63 | 66 | 151 |
| Crum_0912 | glycoside hydrolase family 18 | 153 | 347 | 8 | 46 |
| Crum_1027 | esterase, partial | 1 | 2 | 1 | 0 |
| Crum_1049 | glycosyl hydrolase like GH101 | 244 | 560 | 157 | 384 |
| Crum_1051 | β-galactosidase | 34 | 24 | 68 | 77 |
| Crum_1236 | endoglucanase | 60 | 237 | 206 | 467 |
| Crum_1237 | endoglucanase | 10 | 20 | 10 | 11 |
| Crum_1238 | endoglucanase | 12 | 29 | 13 | 14 |
| Crum_1338 | beta-glucosidase | 470 | 641 | 649 | 443 |
| Crum_1411 | galactosidase | 7 | 20 | 33 | 169 |
| Crum_1412 | galactosidase | 56 | 109 | 256 | 449 |
| Crum_1518 | endoxylanase | 23 | 25 | 36 | 20 |
| Crum_1558 | scaffolding precursor | 27 | 144 | 69 | 428 |
| Crum_1559 | endoglucanase | 50 | 285 | 121 | 623 |
| Crum_1566 | glucosidase | 50 | 48 | 20 | 134 |
| Crum_1590 | cellobiose phosphorylase | 2413 | 4895 | 467 | 3164 |
| Crum_1595 | endoxylanase | 10 | 57 | 22 | 291 |
| Crum_1634 | esterase | 2 | 2 | 4 | 2 |
| Crum_1698 | glycoside hydrolase family 5 | 54 | 116 | 48 | 78 |
| Crum_1862 | endoglucanase | 6 | 11 | 15 | 27 |
| Crum_2009 | esterase | 8 | 32 | 28 | 57 |
| Crum_2011 | type 3a cellulose-binding domain protein | 118 | 453 | 252 | 263 |
| Crum_2015 | glycoside hydrolase family 5 | 61 | 41 | 52 | 119 |
| Crum_2036 | alpha-N-arabinofuranosidase | 119 | 243 | 179 | 401 |
| Crum_2062 | endo-1,4-beta-xylanase | 22 | 255 | 40 | 44 |
| Crum_2063 | endo-1,4-beta-xylanase | 27 | 341 | 43 | 41 |
| Crum_2073 | glycosyl hydrolase | 5 | 123 | 6 | 1048 |
| Crum_2209 | esterase | 1 | 2 | 3 | 3 |
| Crum_2289 | pectate disaccharide-lyase | 16 | 114 | 12 | 20 |
| Crum_2311 | mannanase | 364 | 3574 | 677 | 591 |
| Crum_2321 | pectin methylesterase | 8 | 8 | 7 | 9 |
| Crum_2324 | endoglucanase 1 | 58 | 57 | 73 | 28 |
| Crum_2399 | endo-1,4-beta-xylanase | 122 | 311 | 204 | 674 |
| Crum_2400 | beta-xylosidase | 7 | 22 | 9 | 15 |
| Crum_2428 | glucosidase | 2 | 3 | 3 | 89 |
| Crum_2431 | glucosidase | 3 | 4 | 6 | 9 |
| Crum_2442 | feruloyl esterase III | 12 | 39 | 18 | 28 |
| Crum_2458 | glucosidase | 79 | 140 | 116 | 353 |
| Crum_2557 | glycosyl hydrolase | 17 | 23 | 17 | 37 |
| Crum_2624 | endoglucanase | 334 | 954 | 564 | 1090 |
| Crum_2625 | cellobiohydrolase | 838 | 1324 | 2411 | 3249 |
| Crum_2694 | glycoside hydrolase | 260 | 28 | 29 | 64 |
| Crum_2901 | esterase | 28 | 74 | 16 | 25 |
| Crum_2920 | esterase | 60 | 163 | 29 | 67 |
| Crum_2963 | glucosidase | 3 | 9 | 5 | 4 |
| Crum_2965 | glucosidase | 5 | 11 | 12 | 8 |
| Crum_3006 | pectate lyase | 12 | 83 | 11 | 15 |
| Crum_3011 | endoglucanase | 14 | 24 | 11 | 100 |
| Crum_3158 | endoglucanase | 32 | 70 | 46 | 116 |
| Crum_3248 | pectate lyase | 5 | 18 | 44 | 46 |
| Crum_3314 | alpha-galactosidase | 53 | 63 | 66 | 151 |
| Crum_3318 | polysaccharide deacetylase | 1 | 4 | 1 | 0 |
| Crum_3357 | esterase | 67 | 189 | 38 | 203 |
| Crum_3358 | esterase | 41 | 139 | 42 | 262 |
| Crum_3387 | endoxylanase | 3 | 1 | 1 | 31 |
| Crum_3388 | endoxylanase | 4 | 1 | 1 | 44 |
| Crum_3549 | glycoside hydrolase | 50 | 84 | 104 | 40 |
| Crum_3559 | endoxylanase | 28 | 52 | 6 | 117 |
| Crum_3560 | endo-1,4-beta-xylanase | 2 | 15 | 4 | 23 |
| Crum_3566 | glucosidase | 55 | 43 | 50 | 174 |
| Crum_3567 | arabinofuranosidase | 44 | 27 | 38 | 154 |
| Crum_3568 | endoxylanase | 25 | 13 | 21 | 115 |
| Crum_3569 | beta-galactosidase/beta-glucuronidase | 37 | 18 | 32 | 163 |
| Crum_3570 | glucuronidase | 2 | 3 | 4 | 15 |
| Crum_3583 | beta-galactosidase/beta-glucuronidase | 115 | 19 | 293 | 98 |
| Crum_3620 | endoxylanase | 87 | 190 | 77 | 970 |
| Crum_3742 | beta-glucosidase | 7 | 4 | 19 | 38 |
| Crum_3798 | endoglucanase | 137 | 706 | 99 | 406 |
| Crum_3858 | arabinogalactan endo-1,4-beta-galactosidase | 12 | 22 | 19 | 42 |
| Crum_3859 | galactosidase | 9 | 16 | 23 | 47 |
| Crum_3900 | esterase | 286 | 247 | 97 | 132 |

**Supplementary Table S3.** Fibrolytic activities (mU/mg) in the spent cultures of strain H1 in early- or late-growth phases

|  | Cellobiohydrolase | Endoglucanase | Mannanase | Xylanase | Pectinase |
| --- | --- | --- | --- | --- | --- |
| E-0.5% cellobiose | 0.73±0.06 | 289.23±10.82 | NA | NA | NA |
| E-2% filter paper | 6.81±0.71 | 2881.24±134.98 | NA | NA | NA |
| L-0.5% cellobiose | 0.86±0.09 | 260.28±34.12 | 29.5±1.21 | 112.6±3.45 | - |
| L-2% filter paper | 1.32±0.05 | 587.75±39.87 | 85.7±2.42 | 125.7±4.12 | 42.5±2.21 |
| L-0.05% cellobiose | 3.36±0.43 | 367.45±23.46 | NA | NA | NA |
| L-0.5% xylan | 1.66±0.17 | 1187.22±46.56 | NA | 1032.3±43.2 | NA |

E, early exponential growth culture; L, late exponential growth culture；NA not detected; -, activity below the detection limitation.

**Supplementary figures**


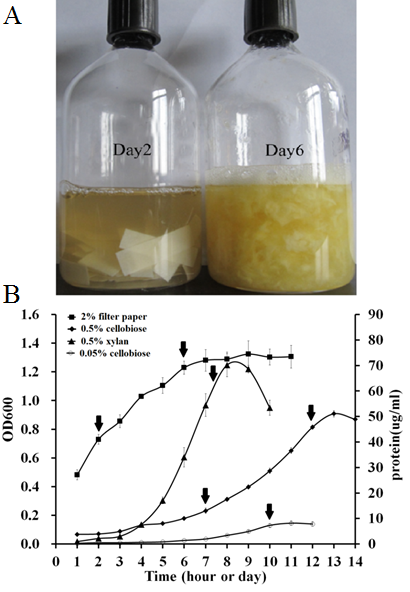


**Supplementary Figure S1. Growth of *Cellulosilyticum ruminicola* H1 in RC medium containing 0.5% cellobiose, 0.05% cellobiose, 0.5% xylan and 2% filter paper.** (A) Degradation of filter paper by *C. ruminicola* H1 at day 2 and day 6 of culture. (B) Growth of *C. ruminicola* H1 on different substrates. The cellular proteins are measured for the filter paper culture, and OD600nm is measured for the remaining cultures. Three batches of culture were incubated at 37℃ under CO_2_. Arrows indicate the time points of RNA extraction for transcriptome analysis.


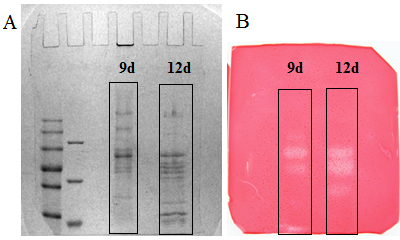


**Supplementary Figure S2. Zymogram assay of cellulose-enriched proteins from the spent filter paper culture.** (A) Cellulose-bound proteins were electrophoresed on SDS-PAGE (8% for separation and 5% for concentration), and visualized by Coomassie brilliant blue staining. Image was acquired by Bio-Rad GelDoc XR+ Image Analysis System. The two framed lanes were cropped and shown in Fig 3A. (B) CMCase activity of cellulose-bound proteins in SDS-PAGE is assayed by an overlaid agarose gel containing CMC and Congo red staining, and image is acquired by Canon PowerShot G11 camera. The two delineated lanes were cropped and shown in Fig. 3B.

**Supplementary Figure S3.**
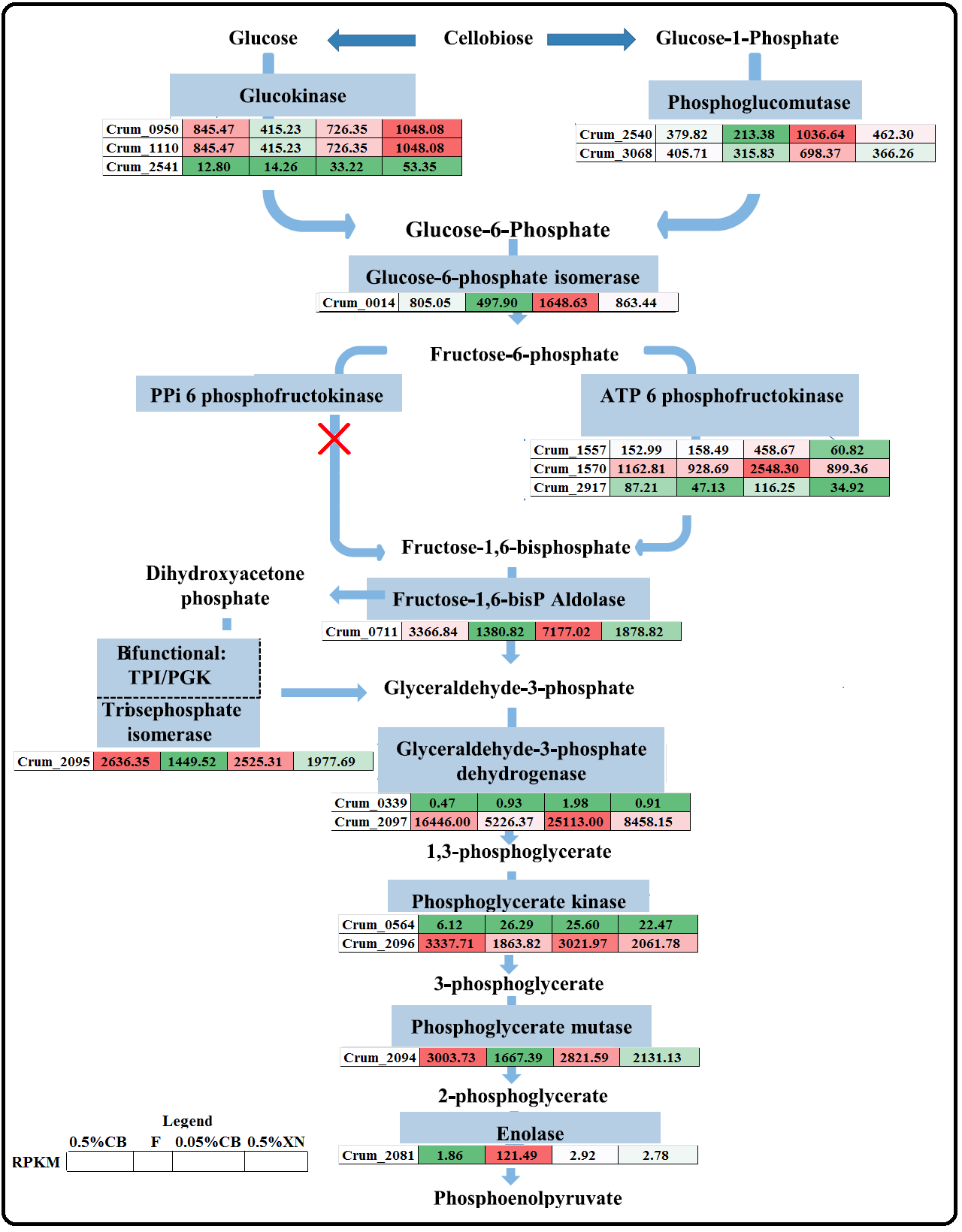
**Differential transcriptions of the genes involved in** Embden–Meyerhof–Parnas (EMP pathway). RPKM values are shown beneath the corresponding genes.
